# Tropa-Cocain in Painless Extraction of Teeth

**Published:** 1894-11

**Authors:** A. D. Hard


ARTICLE VIII.

### TROPA-COCAIN IN PAINLESS EXTRACTION OF TEETH

Tropa-cocain, or benzoyl-pseudo-tropein, as it is scientifically termed, is an alkaloid, first taken from the leaves of the Javanese coca plant by Giesel, of Berlin, through suggestions from Liebermann, who subsequently prepared the same substance synthetically. We owe the credit of its development as a practical and useful therapeutic agent, however, to an American physician, Dr. A. P. Chadbourne, of New York City. My investigations of its usefulness have been confined to the art of painless extraction of teeth. Tropa-cocain is of a somewhat similar nature to cocain, excepting that it is almost free from the toxic and other deleterious effects of that otherwise wonderful drug. It is pre-eminently a local anesthetic, and, as such, it possesses advantages over cocain that make it incomparable. These advantages may be even greater than at present demonstrated. They consist in greater power; quicker action, freedom from systemic disturbances to a great extent; is not depressing to the cardiac motor ganglia; does not produce ischemia or hyperemia; is a moderate antiseptic, rendering subcutaneous injections free from danger of sloughing or abscess; retains its activity for months in solution, and is free from the danger of cocainism, because of its very slight systemic effects.

The principal drawback to its use lies in its present great expense, which is unnecessary, as it is not difficult to obtain, or expensive in manipulation. It is best used in four per cent. solution, using a weak solution of pure sodium chlorid as a menstrum. Its permanency in solution is probably due to lack of fungoid degeneration, as a result of a natural antiseptic quality which cocain does not possess. In the painless extraction of teeth I have been charmed by its use. The many concurrent and subsequent ill effects of cocain have driven it almost completely from the hands of conscientious physicians and dentists. Tropacocain now is offered as a more worthy and reliable substitute. By its use we may assure the possessor of unwanted teeth that extraction of offending molars may be compared to clipping of the finger-nails—nothing more. We may coolly secure a firm base hold of a decayed crown and successfully remove what would otherwise more than likely to be broken, bungled and blotched. We may even crush through the edge of the alveolar process, grasp an ugly root, and smile as it quickly and without pain emerges into the light of decay. My experience has taught me that details must not be neglected, however, in its use, if we would have the best results. The gums near the teeth to be extracted should be first wet with the solution, and after a delay of about one minute, two minims should be injected with a hypodermic syringe, in two places about half inch apart, and not less than one-fourth inch from the margin of the gums, both inside and outside of the teeth, making four injections of two minims each. This amount is sufficient for the extraction of from one to six teeth in that vicinity. Its effect will continue about twenty minutes. Minims should be gaged by the small thumb screw on the piston of the syringe. The injections should be deep, and in the direction of the roots.—A. D. HARD, M. D., in *Medical World.*

